# Yeast Deletomics to Uncover Gadolinium Toxicity Targets and Resistance Mechanisms

**DOI:** 10.3390/microorganisms11082113

**Published:** 2023-08-19

**Authors:** Nicolas Grosjean, Marie Le Jean, Jordan Ory, Damien Blaudez

**Affiliations:** 1 DOE Joint Genome Institute, Lawrence Berkeley National Laboratory, Berkeley, CA 94720, USA; ngrosjean@lbl.gov; 2 Université de Lorraine, CNRS, LIEC, F-57000 Metz, France; marie.lejean@univ-lorraine.fr; 3 Université de Lorraine, CNRS, LIEC, F-54000 Nancy, France; jordan.ory@live.fr

**Keywords:** lanthanides, functional toxicogenomics, GBCA, metal homeostasis

## Abstract

Among the rare earth elements (REEs), a crucial group of metals for high-technologies. Gadolinium (Gd) is the only REE intentionally injected to human patients. The use of Gd-based contrasting agents for magnetic resonance imaging (MRI) is the primary route for Gd direct exposure and accumulation in humans. Consequently, aquatic environments are increasingly exposed to Gd due to its excretion through the urinary tract of patients following an MRI examination. The increasing number of reports mentioning Gd toxicity, notably originating from medical applications of Gd, necessitates an improved risk–benefit assessment of Gd utilizations. To go beyond toxicological studies, unravelling the mechanistic impact of Gd on humans and the ecosystem requires the use of genome-wide approaches. We used functional deletomics, a robust method relying on the screening of a knock-out mutant library of *Saccharomyces cerevisiae* exposed to toxic concentrations of Gd. The analysis of Gd-resistant and -sensitive mutants highlighted the cell wall, endosomes and the vacuolar compartment as cellular hotspots involved in the Gd response. Furthermore, we identified endocytosis and vesicular trafficking pathways (ESCRT) as well as sphingolipids homeostasis as playing pivotal roles mediating Gd toxicity. Finally, tens of yeast genes with human orthologs linked to renal dysfunction were identified as Gd-responsive. Therefore, the molecular and cellular pathways involved in Gd toxicity and detoxification uncovered in this study underline the pleotropic consequences of the increasing exposure to this strategic metal.

## 1. Introduction

Gadolinium (Gd) belongs to the rare earth elements (REEs), which are sub-divided as light or heavy REEs according to their atomic mass [1]. Gd is the first heavy REE in the lanthanide series and one of the most abundant heavy REEs in the Earth’s crust [2]. To supply its use for various applications, the production of Gd increased over the last two decades [3,4]. Amongst those applications, Gd is employed in the fabrication of lasers, magnets, cathodic ray tubes and different superconducting alloys [5]. More importantly, due to its paramagnetic properties, this metal is mainly used in imaging medicine [6,7].

In an aqueous solution, Gd adopts a trivalent state (Gd^3+^) and is highly toxic for organisms due to its competition with Ca in Ca-dependent cellular processes [8]. For these reasons, Gd is complexed with linear or macrocyclic chelators for its use in medicine as a contrast agent for magnetic resonance imaging (MRI). These Gd-based contrasting agents (GBCAs) are less toxic and are normally excreted from the human body via the urinary system [9]. However, the urine of patients undergoing MRI is not treated and therefore ends up in wastewater systems. An EU-wide monitoring survey in wastewater treatment plants reported that Gd was one of the most abundant compounds among a list of over 200 emerging contaminants [10]. Another environmental study revealed that the percentage of anthropogenic Gd varies from 47 to 93%, showing that this element is dominantly influenced by urban sewage and hospital/industrial activities [11]. Since Gd is not sorted by wastewater plants, this element migrates to rivers and oceans as reported by several studies showing abnormal concentrations in these ecosystems [12,13,14] reaching up to 409 ng/L at the vicinity of an underwater outfall [15]. Furthermore, it has been identified that invertebrate aquatic species are impacted by Gd at different biological levels, including alterations in gene expression, cellular homeostasis, shell formation, metabolic capacity and antioxidant mechanisms [15].

One of the main concerns with Gd resides in its impact on human health. GBCA compounds can trigger adverse drug reactions (ADRs), which have been estimated to occur in 0.07 to 2.4% of patients [16]. Even if non-renal ADRs occur, the majority of GBCA-mediated side effects concerns nephrogenic systemic fibrosis associated with the impaired excretion of GBCAs in subjects with kidney diseases [17]. Since the prevalence of Gd seems to drive fibrosis in the context of renal dysfunction, nephrogenic systemic fibrosis has been renamed Gd-associated systemic fibrosis [18]. Such ADRs correlate with the instability of Gd^3+^ complexes, leading to the release of Gd ions in vivo [19]. Indeed, while most GBCAs are almost exclusively excreted by passive glomerular filtration through the kidney (without secretion nor reabsorption) or some with little hepatic uptake [20,21], Gd ions can be displaced from these complexes and bind to phosphate and carbonate groups, creating insoluble precipitates that are deposited in different organs and tissues [22]. Gd deposition has been reported as not being specific to kidneys, but rather can occur in other organs. In subjects with kidney diseases and healthy individuals, Gd deposition has been found in bones after a single injection [23] as well as in the brain of subjects with normal renal function [24,25]. Following these findings, the US FDA announced a new class warning and required new safety measures for all GBCA compounds [26], while the European Medicines Agency issued restrictions and suspensions for linear agents containing Gd [27].

Although clinical cases of Gd-induced disorders/diseases are documented, and gadolinium-toxicity assays have been performed on various organisms [28,29], little information is available on the interaction of Gd ions with eukaryotic cells. The toxicity of Gd^3+^ can be partly due to its ability to mimic divalent cations, specifically Ca^2+^ [17]. Indeed, given their similar ionic radii (1.06 Å for Ca^2+^; 1.00 Å for Gd^3+^) and metal coordination chemistry (6–8 ligands), REEs display strong chemical similarities with ions such as Ca^2+^ [30,31]. Consequently, Gd has often been used, as well as other light REEs, as an antagonist to study Ca fluxes in cells because Gd is an excellent calcium channel blocker. Additionally, Gd, along with other REEs, has been shown to replace Ca^2+^ with greater affinity in a number of Ca-binding proteins. However, other cellular targets of Gd-mediated toxicity remain mostly unknown, while the molecular mechanisms sustaining Gd tolerance are still to be discovered.

Functional deletomics is a genome-wide-based approach comparing the phenotypic response of knock-out mutants with that of the wild-type to infer the relationships between genes and the level of tolerance to various stressors. This strategy has been deployed by numerous studies, notably to uncover toxicity and/or tolerance mechanisms in *Saccharomyces cerevisiae* upon exposure to numerous transition metals [32,33,34,35]. Yeast is one of the best-characterized eukaryotic organisms and shares many cellular pathways and functions with humans [36,37]. Such deletomic approaches in yeast can therefore not only expand our knowledge regarding the cellular mechanistic response, but also allow for the exploration of potentially conserved toxicity responses in humans. A recent study conducted by Pallares et al. described the library screening of *S. cerevisiae* using low toxic concentrations of Gd using a pool screening approach [38]. In the present study, we employed a different but complementary approach that allows for the identification of Gd-sensitive and Gd-resistant mutants by screening the entire arrayed collection of the BY4741-derived knock-out mutants for Gd stress using sub-lethal concentrations. Such arrayed screening and the longer Gd exposure allowed for the identification of a broader set of functions related to Gd stress. The data were compared to those obtained from other metal deletomic screens performed with other metals or chemical stressors and allowed for the discovery of not only metal-common responses, but also Gd-specific responses. The cellular compartments and functions involved in the response to Gd stress were investigated in detail. In conclusion, the results shed light onto the molecular mechanisms involved in Gd detoxification as well as those related to Gd-triggered toxicity in this model organism. These data will provide the basis for better understanding Gd impact on environmental organisms and human health.

## 2. Materials and Methods

### 2.1. Yeast Strains, Growth Medium and Chemicals

The haploid BY4741 (MATa; *his3*Δ1 *leu2*Δ0 *met15*Δ0 *ura3*Δ0) wild-type strain of *Saccharomyces cerevisiae* as well as its related library of 4733 knock-out deletion mutants (EUROSCARF) were used in this study. Gadolinium (III) chloride hexahydrate (99.999% purity, #203289) was obtained from Sigma-Aldrich (Saint-Quentin-Fallavier, France).

### 2.2. Identification of Gd-Sensitive and Gd-Resistant Mutants

**Figure 1:**
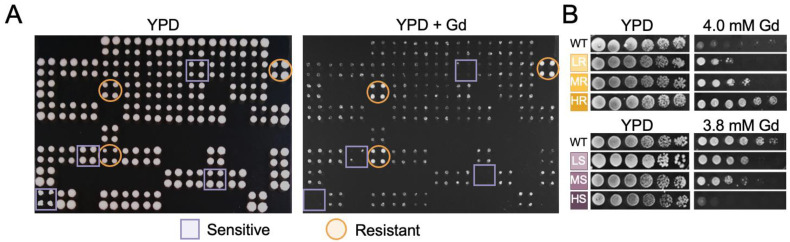
Representative plates of yeast mutants grown in absence (YPD) and presence of sub-toxic concentrations of gadolinium (YPD + Gd). (**A**) Representative 384-well format growth test for the primary screen. For each mutant, four replicates were performed (represented in colored squares or circles). An example of mutants identified as resistant are indicated by orange circles, while mutants identified as sensitive are indicated by purple squares. (**B**) Representative pictures of phenotypes from the validation screen. Yeast phenotype to gadolinium was tested using 3.8 mM to determine sensitive mutants and 4.0 mM to determine resistant mutants. Mutants were ranked as “high” (HS), “medium” (MS), or “low” (LS) sensitivity to gadolinium when displaying an absence of growth at the first, second–third or fourth–fifth dilutions, respectively. Mutants were ranked as “low” (LR), “medium” (MR), or “high” (HR) resistant to gadolinium when displaying growth at the second, third–fourth, or fifth–sixth dilutions, respectively.

The wild-type strain and the deletion mutants were grown at 28 °C until the stationary phase in 200 µL of YPD medium (10 g·L^−1^ peptone, 20 g·L^−1^ glucose and 10 g·L^−1^ yeast extract) in 96-well master plates. Mutants sensitive or resistant to Gd were identified by comparing their growth to that of the wild-type on YPD medium containing 3.9 mM Gd. The strains were pin replicated using a Thermo Scientific™ Nunc™ (Illkirch-Graffenstaden, France) Replication System (250520). Four technical replicates were performed for each mutant. Growth was recorded after 5 days at 28 °C. Mutants with no growth on the Gd-supplemented medium were determined as sensitive (Figure 1). Mutants for which growth was not affected compared to that on the Gd-free medium were assigned as resistant.

### 2.3. Phenotypic Confirmation of Mutants

The phenotypes of Gd-sensitive and Gd-resistant mutants were individually confirmed by serial dilution spot assays. Mutants were grown as previously described, and serial tenfold increment dilutions were performed. Five microliters of six ten-fold dilutions were spotted on the same medium containing 3.8 mM Gd to verify Gd-sensitive mutants or 4.0 mM Gd for Gd-resistant mutants. These two concentrations were chosen to allow for the discrimination of sensitive and resistant mutants relative to the wild-type strain. A concentration of 3.8 mM was the highest concentration of Gd at which we did not observe a growth defect of the wild-type strain at each dilution, while 4.0 mM was the highest concentration at which we still observed a growth of the wild-type strain. Phenotypes were analyzed after 5 days of growth at 28 °C. Sensitivity and resistance levels were assigned to the mutants according to the number of dilutions where cells grew. Consequently, mutants exhibiting a reduction in colony-forming ability at the first, second–third or fourth–fifth dilutions were classified as “high” (HS), “medium” (MS) or “low” (LS) sensitive, respectively. Conversely, mutant strains exhibiting an increase in colony-forming ability at the first–second, third–fourth, or fifth–sixth dilutions were classified as “low” (LR), “medium” (MR), or “high” (HR) resistant, respectively.

For the phenotypic characterization of resistant mutants exposed to different REEs (from La to Lu), we selected the highest REE concentration at which we observed growth of the wild-type strain, corresponding to 4.2 mM La, 4.2 mM Ce, 6.7 mM Pr, 4.5 mM Nd, 4.6 mM Sm, 4.0 mM Eu, 4.0 mM Gd, 3.9 mM Tb, 3.6 mM Dy, 3.9 mM Ho, 3.9 mM Er, 3.6 mM Tm, 3.6 mM Yb and 3.6 mM Lu. For the phenotypic characterization of sensitive mutants, we selected the highest concentration at which we did not obverse a growth defect of the wild-type strain, corresponding to 4.0 mM La, 3.8 mM Gd and 3.6 mM Yb.

### 2.4. Data Analyses

Human homolog(s) of *S. cerevisiae* genes and any of their associated OMIM disease phenotypes were obtained from the SGD YeastMine platform (http://yeastmine.yeastgenome.org/yeastmine/, accessed on 30 June 2021). Cellular compartment and biological process analyses were performed with clusterProfiler [39] and evaluated for statistical significance (cut-off: *p*-value < 0.05). Clustering (heatmaps) was performed using the pheatmap package in R. Gd-sensitive and Gd-resistant mutants identified in the present study were compared with those from other screens carried out on yeast KO mutant libraries on Al [40,41], As [34,35,42,43], Cd [32,35,43,44], Co [45], Cr [35,46], Cu [33,35,45,47], Fe [33,45,47], Mn [45,47,48], La [49], Nd [50], Ni [32,45,51], Zn [35,45,47,52], Y [53], Yb [49] and other stressors [54,55,56,57,58]. When several independent studies performed similar screenings on the same stressor, the corresponding lists of mutants were combined.

## 3. Results and Discussion

### 3.1. Genomic Phenotyping of Gadolinium Toxicity

The entire knock-out mutant library of *S. cerevisiae* BY4741 was screened under sub-lethal concentrations of Gd to identify mutants displaying a modified growth relative to the wild-type strain (Figure 1A, Supplementary Table S1). Such a sub-lethal threshold selection is typically used in metal toxicity screening. These concentrations can be encountered in the environment, notably at microscale (e.g., soil particles and tissues) where special and chemical heterogeneity can be found [59,60]. Following the first screening, a confirmation screen was performed to eliminate false positives (Figure 1B).

**Figure 2:**
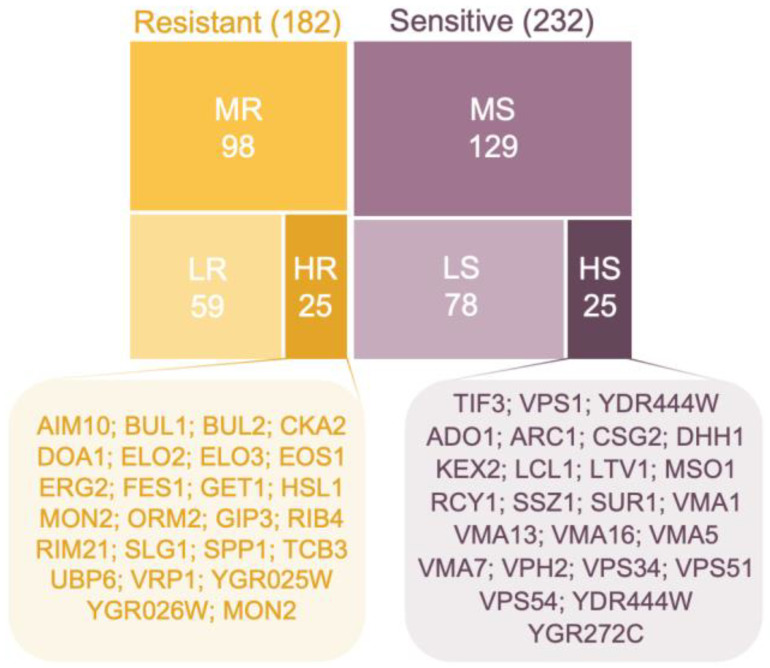
Distribution and ranking of mutants displaying a modified growth phenotype towards gadolinium compared to the wild-type strain. The proportion of resistant mutants is depicted by the yellow boxes, while the proportion of sensitive mutants is depicted by the purple boxes. The number of mutants in each low (LR-LS), medium (MR-MS) and high (HR-HS) categories is shown, and ORF names of the highly resistant (HR) and highly sensitive (HS) mutants are shown.

Following the verification screen, a total of 414 mutants were confirmed, with 182 resistant (increased tolerance to Gd) and 232 sensitive (lower tolerance to Gd) mutants (Figure 2). Here, we identified a similar number of sensitive mutants than what was found by Pallares et al. [33]. While they identified only a limited number of resistant mutants to Gd, our results provide an additional value, with three times more resistant mutants. The 414 mutants were subsequently discriminated into six categories, ranging from highly sensitive (HS) to highly resistant (HR) according to their tolerance level to Gd. Similar proportions of mutants, whose disrupted ORF is displayed in Figure 2, exhibited the highest resistance to Gd (25 mutants, 13.7% of resistant mutants) and the highest sensitivity (25 mutants, 10.8% of sensitive mutants). More than 50% of resistant and sensitive mutants were categorized as medium resistant (MR, 98 mutants, 53.8%) and medium sensitive (MS, 129 mutants, 55.6%), and more than 30% were categorized as low resistant (LR, 59 mutants, 32.4%) and low sensitive (LS, 78 mutants, 33.6%).

### 3.2. Cellular Hotspots for Gd Toxicity and Resistance

**Figure 3:**
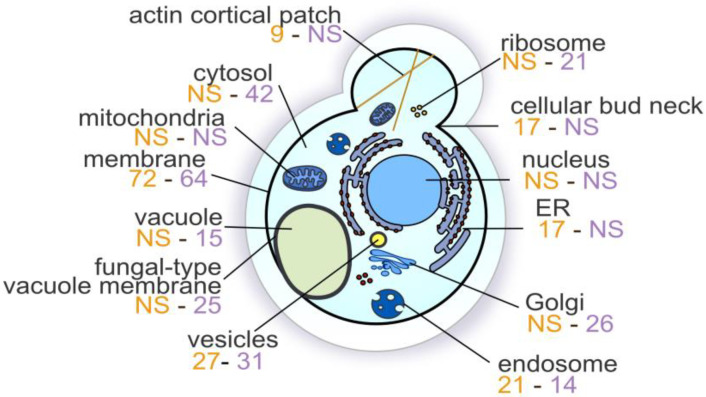
**Cellular compartments involved in a gadolinium stress in *Saccharomyces cerevisiae*.** Values in orange and in purple are the number of resistant and sensitive mutants, respectively, when the compartment was significantly represented. NS: Not significant. Cellular compartment analysis was performed with clusterProfiler [39] and evaluated for statistical significance (cut-off: *p*-value < 0.05).

We first approached the analysis of the obtained set of mutants by looking at the enriched cellular compartments, providing insights into the cellular hotspots for both Gd toxicity and resistance. Cellular compartments affected by mutations enriched in the Gd screen were retrieved by GO-term enrichment analysis (Figure 3 and Supplementary Table S2).

These compartments represent key elements in either Gd toxicity or resistance. Cellular compartments, such as the membrane (136 mutants, *p*-value = 0.00045) and endosomes (35 mutants, *p*-value = 2.46 × 10^−13^), were represented in both sets of resistant and sensitive mutants. Rare earth elements have been previously shown to trigger endocytosis in plant cells [61,62], and endocytosis has been linked to other rare earth elements in yeast [49,53]. Additionally, rare earth elements have been shown to reduce membrane fluidity [63] and increase membrane permeability [64,65]. More specific compartments were particularly enriched either in sets of resistant mutants or sensitive mutants. In sensitive mutants, the Golgi apparatus (26 mutants, *p*-value = 3.14 × 10^−9^), the GARP complex (3 mutants, *p*-value = 0.0042), the cytosol (42 mutants, *p*-value = 0.0019) and ribosomes (21 mutants, *p*-value = 0.00015) were the most significantly impacted. Additionally, the vacuole (15 mutants, *p*-value = 0.0013), the fungal-type vacuolar membrane (25 mutants, *p*-value = 1.62 × 10^−9^) and, more precisely, the vacuolar proton-transporting V-type ATPase, V1 domain (6 mutants, *p*-value = 3.27 × 10^−5^) were enriched. The vacuole is an essential compartment for the storage of toxic metals, such as Cd and Zn, to prevent any toxic effect of free metals in the cytosol by interacting with proteins. Resistant-specific compartments that were impacted essentially represented actin cortical patches (9 mutants, *p*-value = 0.00086), ESCRT-I complex (4 mutants, *p*-value = 0.00015) and cellular bud neck (17 mutants, *p*-value = 0.00067).

### 3.3. OMIM Human Orthologs and Diseases

GBCA compounds were linked to both renal- and non-renal-associated adverse reactions in up to 2.4% of patients [16]. The large predominance of these cases involves nephrogenic systemic fibrosis, which is associated with the impaired excretion of GBCAs in subjects with kidney diseases [17]. Through the interrogation of the OMIM human-related diseases tool from the SGD yeastMine platform, we identified one or more human orthologs for the 236 genes disrupted in mutants with modified phenotype towards Gd (Supplementary Table S1). Amongst these, 68 were associated with diseases or genetic disorders, such as cancers, and renal dysfunction. Related to the latter, the disruption of *VMA2*, which encodes for a subunit of the vacuolar ATPase, leads to a medium sensitivity (MS) to Gd. Both human orthologs of yeast *VMA2*, namely ATP6V1B2 and ATP6V1B1, have been linked to renal tubular acidosis. Similarly, *LST7* (low sensitive mutant), which is an ortholog of the human folliculin, is related to renal cell carcinoma. Another sensitive strain, mutated for VPS33, is an ortholog of VPS33B and VPS33A, both of which are linked to arthrogryposis, renal dysfunction and cholestasis 1 (ARCS1). Finally, a mutation of *GZF3*, a GATA zinc finger transcription repressor, is resistant to Gd. The human ortholog of *GZF3* is linked to many disorders, including HSDR (hypoparathyroidism, sensorineural deafness and renal disease). Gd deposition in other organs has also been reported in humans displaying both renal dysfunction and normal renal function. Gd deposition was found not only in bones after the administration of a single dose [23], but also in the brain [24,25]. Other yeast genes found in our Gd screen were linked to micronutrient homeostasis defect and a bone-related condition. For the latter, the human homologs of *SAC6* (PLS3, LCP1 and PLS1), for which mutations in yeast leads to Gd resistance, were associated with bone mineral density diseases through quantitative trait locus analysis (BMND18). Finally, we identified mutants for genes orthologs to human genes linked to anemia, therefore perturbating iron homeostasis. Three of these encoded proteins were SMF1 and SMF2 (medium and low sensitivity, respectively), which are involved in divalent cations transport across membranes and HEM25 (medium resistance), belonging to the mitochondrial solute carrier family. Identifying these predisposition factors linked to renal pathologies and other diseases provides further information on the potential stronger impact of Gd and GBCAs on predisposed individuals. Furthermore, the delineation of genes and processes not previously linked to known GBCA-related pathologies also offers insights into targets of interest for further strategies to prevent adverse reactions caused by GCBA.

### 3.4. Gd Deletomics Profile Comparison to Other Metals

**Figure 4:**
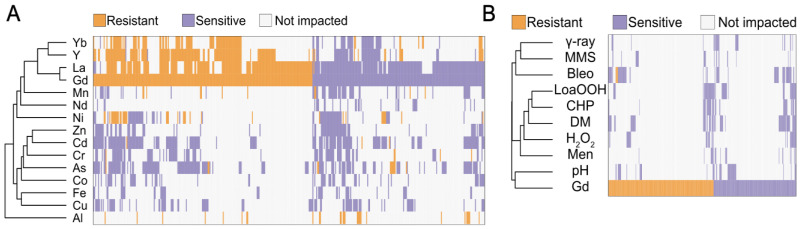
(**A**) In silico comparison of phenotypes of the mutants identified in this study compared to those from other metal-based screening studies. (**B**) Hierarchical clustering of gadolinium sensitivity or resistance-conferring mutations with the mutant sensitivity/resistance profiles of other stressors. The *x*-axis corresponds to gene deletions, and the *y*-axis represents the different elemental or physico-chemical stressors. Mutant strains exhibiting either a higher sensitivity, a higher resistance or no phenotype change when compared to wild-type are shown in purple, orange and gray, respectively. Metal and non-metal stressors were selected from previous genomic phenotyping screenings conducted on deletion mutant collections. Methyl methane sulfonate (MMS), gamma-radiation (γ-ray), alkaline pH (pH), menadione (Men), hydrogen peroxide (H_2_O_2_), cumene hydroperoxide (CHP), linoleic acid 13-hydroperoxide (LoaOOH) and diamide (DM). Hierarchical clustering was performed with the following parameters: average linkage and uncentered correlation. Clusterings were performed using the pheatmap package.

To further assess the singularity of the cellular impact of Gd compared to other metals, we performed a clustering analysis with other genome-wide deletion mutant library screenings not only on monovalent (Cu), divalent (Cd, Co, Cr, Zn, Ni and Mn) and trivalent (Al and Fe) cations, but also on the metalloid As, and other rare earth elements (Y, La, Yb and Nd), all performed in similar conditions (Figure 4A). Despite several biases, including the focus of most other studies on sensitive mutants, several observations can be made: (i) There is a strong correlation between Gd and other rare earth elements profiles, specifically with La, a light rare earth element. Nevertheless, we can denote a few mutants displaying an opposite phenotype to the two rare earth elements (sensitive to Gd while resistant to Y and/or Yb). (ii) Mn, Ni and the rare earth element Nd are also clustered together with Gd. (iii) Most of the mutants found to be resistant towards Gd, La, Yb, Y, Ni and Al are sensitive to other elements. A similar analysis was performed to compare the deletomic profile of Gd to genome-wide screens on chemical stressors, including oxidative stress, pH and γ-rays (Figure 4B). In this analysis, Gd profile was clustered with an alkaline pH. Oxidative stress was previously reported as the main toxic effect of REEs but was not clustered with Gd in this analysis, similarly to other REEs (La, Yb and Y) [49,53].

### 3.5. Vacuolar Acidification Is Essential under Gd Stress

**Figure 5:**
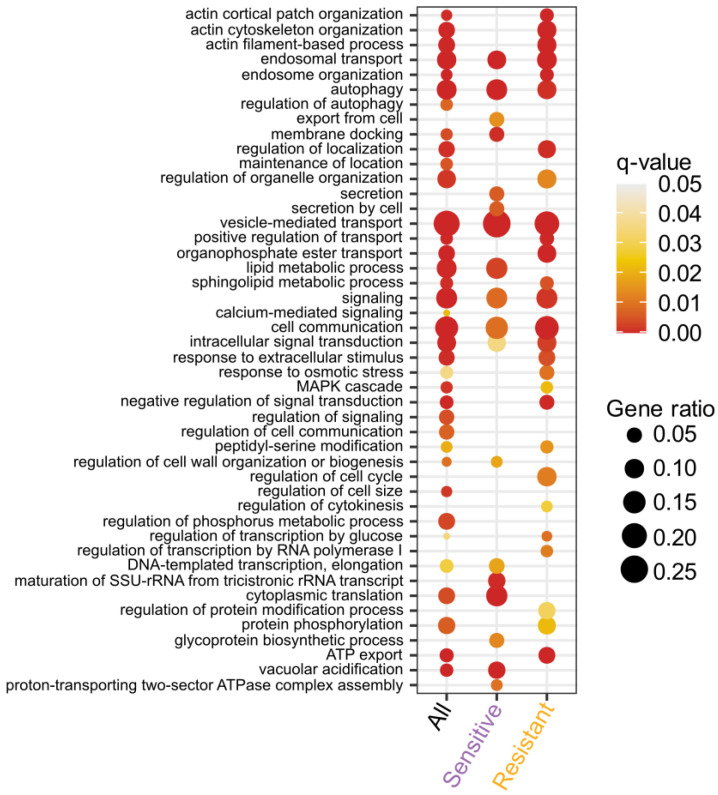
GO-term analysis of the biological pathways (BP) enriched in the mutants identified from the library screening for gadolinium response. The most significant functions enriched were identified by separating the mutants that are sensitive and resistant to gadolinium or by combining them (All). Biological process analysis was performed with clusterProfiler [39].

To harness the biological pathways that play pivotal roles in either the sensitivity or resistance mechanism to Gd, we used the entire set of mutants displaying a modified fitness towards Gd for a GO-term enrichment analysis (Figure 5, Supplementary Tables S3 and S4). Hence, to understand biological pathways involved in resistance and Gd detoxification, we focused on mutants exhibiting a sensitive phenotype (i.e., if a gene is required for resistance, its deletion will lead to sensitivity of the strain) which were enriched in six main GO-term pathways (and related GO-terms).

**Figure 6:**
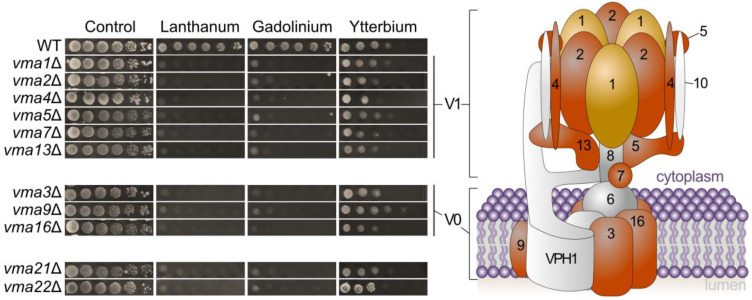
Growth of yeast mutants knocked out for the vacuolar membrane ATPase complex (VMA) involved in the acidification of the vacuole in response to toxic concentrations of lanthanides. Yeast growth was assessed on YPD medium without lanthanides (control) or supplemented with 4.0 mM La, 3.8 mM Gd or 3.6 mM Yb, with 10-fold serial dilutions of cultures from left to right in each panel. A representative plate (out of 3 independent experiments) is shown. Plates were incubated for 5 days at 30 °C. A schematic representation of the VMA complex with its different subunits (numbers depict the subunit number, e.g., 1 represents subunit Vma1p) is shown. Subunits colored in orange/yellow depict the sensitive phenotype of the mutants, while grey color describes the absence of phenotype in the screen performed.

First, GO-terms related to vacuolar acidification were represented by 12 mutants (*p*-value = 1.5057 × 10^−10^). Furthermore, it includes mutants for genes encoding V-ATPase subunits, such as *VMA21* and *VMA22*, or V-ATPase assembly factors, such as *PKR1* and *VPH2*. Mutants for most of the V-ATPase subunit-encoding genes were either low (*VMA4*), mild (*VMA2*, *VMA3*, *VMA9*, *VMA21* and *VMA22*) or highly (*VMA1*, *VMA5*, *VMA7*, *VMA13* and *VMA16*) sensitive to Gd. Interestingly, while this implication of VMA genes was not identified in another study [38], we confirmed that this sensitive phenotype also occurs when exposed to La but not to the heavy REE Yb (Figure 6). The VMA complex ensures the proper acidification of the vacuole [66]. Therefore, we can argue that Gd and La impact the vacuolar pH, in which case, the absence of a functional VMA complex has a detrimental effect on cell viability. One hypothesis is that La and Gd are being sequestrated in the vacuole, either as REE complexes or as cations, while heavier REEs would not.

### 3.6. Maintenance of Glycoproteins, Cell Wall Organization and Biogenesis Is Essential under Gd Stress

The cell wall organization and biogenesis pathways were significantly enriched together with the glycoprotein-related pathway in the pool of sensitive mutants (Figure 5 and Supplementary Table S3). Mutants for *SAC7* and *SLT2* were sensitive to Gd. These genes encode for a GTPase-activating protein for Rho1p and a serine/threonine MAP kinase, respectively, both of which are shown to be involved in regulation of cell wall integrity [67]. Additionally, mutants for genes involved in glycoprotein biosynthesis were also identified. More specifically, these strains were mutated for genes encoding for glycosyltransferases (*ALG6* and *ALG8*), mannosyltransferase (*MNN9*, or dolichyl pyrophosphatase (*CAX4*), which ensure the proper biogenesis and glycosylation of essential components of the cell wall [68]. While ALG8 has been identified in a previous study [38], the other mutants identified here strengthen the role of the cell wall to Gd exposure. The central role of the cell wall in the interaction with REEs has been studied with the two distinct REEs La and Yb, for which opposite phenotypes were observed [49]. As Gd is in the middle of the lanthanide series, it is reasonable to assume that the mild phenotype observed towards Gd is because of its intermediate chemistry, between the two extremes lanthanides, La and Yb.

### 3.7. Mutations in Genes Involved in Lipid Metabolic Process Account for Gd Sensitivity

In yeast, the membrane is the second interface following the cell wall. Defects in phospholipid metabolic processes led to sensitivity to Gd (Figure 5 and Supplementary Table S3). Eleven mutants, enriched under this biological process, were sensitive to Gd. Five out of the eleven mutants are related to phosphatidylinositol metabolism. Phosphatidylinositol is an essential lipid preponderant in the membrane with functions ranging from membrane fluidity to signaling [69]. Phosphatidylinositol-4-phosphate (PI4P) accumulation has been shown at the plasma membrane of *Arabidopsis* cells triggered by La and Gd [70]. The accumulation of these negatively charged stress-specific phosphoinositides at the plasma membrane is involved in the rearrangement of the endoplasmic reticulum–plasma membrane contact site complexes, which are needed for ER stress response and adaptation to environmental cues. Additionally, phosphoinositides have roles in autophagy and endocytic vesicular trafficking [69].

### 3.8. Actin Cytoskeleton and Endocytosis Are Major Targets of Gd Toxicity

**Figure 7:**
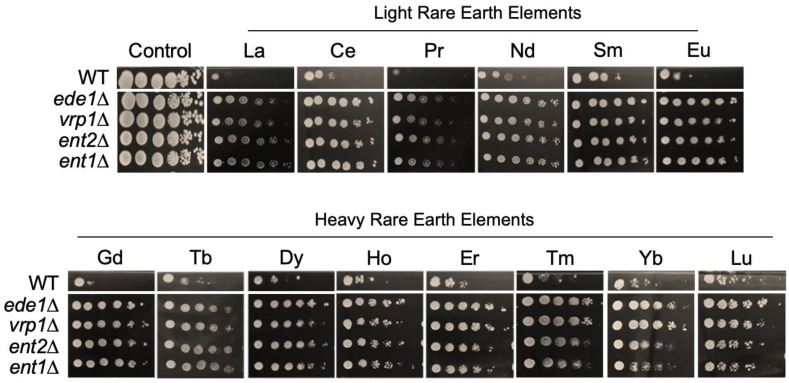
**Growth of mutants involved in the endocytosis pathway in response to toxic concentrations of lanthanides.** Yeast growth was assessed on YPD medium without lanthanides (control) or supplemented with 4.2 mM La, 4.2 mM Ce, 6.7 mM Pr, 4.5 mM Nd, 4.6 mM Sm, 4.0 mM Eu, 4.0 mM Gd, 3.9 mM Tb, 3.6 mM Dy, 3.9 mM Ho, 3.9 mM Er, 3.6 mM Tm, 3.6 mM Yb, or 3.6 mM Lu, with 10-fold serial dilutions of cultures from left to right in each panel. A representative plate (out of three independent experiments) is shown. Plates were incubated for 5 days at 30 °C.

To explore the biological pathways pertaining to the sensitivity of yeast cells to Gd, we performed a GO-term analysis specifically on mutants displaying resistance to Gd (Figure 5 and Supplementary Table S3). Several intertwined biological pathways were enriched. Because two of the major functions fulfilled by the actin cytoskeleton are endocytosis and vesicle trafficking [71], this could explain why several related GO-terms were enriched, such as actin cytoskeleton organization, endosomal and vesicle-mediated transport. As shown for plant protoplasts, for which rare earth elements were able to trigger endocytosis [61], our findings suggest an involvement of endocytosis in yeast response not only to Gd, but also to all rare earth elements (Figure 7).

Cr was previously demonstrated to trigger endocytosis in *S. cerevisiae*, which was first proposed to explain the sensitivity of endocytosis mutants ∆Sac6p, ∆Chc1p or ∆End3p that harbored a higher Cr content [72]. It was suggested that the endocytic inactivation of Cr transporters at the plasma membrane in an ubiquitin-dependent manner is an essential pathway to cope with Cr toxicity [72]. However, Gd seems to rely on a different mechanism. Interestingly, others concluded that the disruption of endocytosis was deleterious to yeast cells in response to Gd, but none of the mutants we identified were found in their study [38]. This comparison supports the importance of performing genome-wide library screenings using different concentrations. Here, endocytosis mutants, such as *end3*∆, *ede1*∆, *vrp1*∆, *she4*∆, *sla1*∆, *ent1*∆, *ent2*∆, *ent4*∆ and *ent5*∆, were resistant to Gd and to all other REEs (Figure 7). Vrp1p and End3p mutants are known to take part in endocytosis, cytoskeletal organization, cytokinesis (Vrp1p) and cell wall morphogenesis (End3p) [73]. She4p is required for the proper localization of the type I myosin Myo5p and F-actin [74], while Ent2p, Ent4p and Ent5p act at different stages of endocytosis. Their resistance infers that endocytosis is involved in REE toxicity, possibly by mediating intracellular REE uptake. The hypothesis of yeast plasma membrane efflux transporters being regulated by endocytosis, thereby modulating the intracellular uptake of metals, can also be considered. Such post-translational regulation by endocytosis has been shown for Zrt1p and Ctr1p, (Zn and Cu uptake transporters, respectively) which are further degraded in the vacuole [75,76,77]. Therefore, endocytosis inhibition could favor the accumulation of a putative REE efflux transporter at the plasma membrane, allowing for REE detoxification outside the cell. Yet, persistence of efflux transporters at the plasma membrane would be balanced by the persistence of influx transporters.

### 3.9. Disruption of Vesicle Trafficking Renders Yeast Cells Resistant to Gd

**Figure 8:**
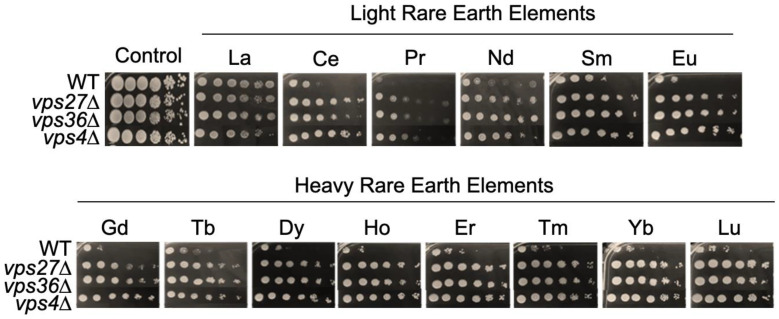
Growth of mutants involved in the ESCRT/GARP/Retromer complex pathway in response to toxic concentrations of lanthanides. Yeast growth was assessed on YPD medium without lanthanides (control) or supplemented with 4.2 mM La, 4.2 mM Ce, 6.7 mM Pr, 4.5 mM Nd, 4.6 mM Sm, 4.0 mM Eu, 4.0 mM Gd, 3.9 mM Tb, 3.6 mM Dy, 3.9 mM Ho, 3.9 mM Er, 3.6 mM Tm, 3.6 mM Yb or 3.6 mM Lu, with 10-fold serial dilutions of cultures from left to right in each panel. A representative plate (out of three independent experiments) is shown. Plates were incubated for 5 days at 30 °C.

Closely related to endocytosis, the ubiquitin-dependent protein catabolic process via the MVB (Multi Vesicular Body) pathway was also found to be enriched (Figure 5, Supplementary Table S3). Thirteen mutants linked to this pathway displayed resistance to Gd. The prevacuolar compartment (PVC) and MVB formations ensured by the ESCRT complex are pathways that allow for the recycling of misfolded membrane proteins (e.g., membrane receptors and transporters) as well as vacuolar components, or the elimination of plasma membrane regions through pinocytosis [78]. Mutants for the entire ESCRT machinery were resistant, including ESCRT-0 (ΔVps27), ESCRT-I (ΔStp22, ΔVps28, ΔSrn2 and ΔMvb12), ESCRT-II (ΔSnf8, ΔVps25 and ΔVps36) and ESCRT-III (ΔDid4, ΔVps20 and ΔVps24) [78] (Figure 8, Supplementary Table S1). ∆Vps4, which catalyzes ESCRT-III disassembly and subsequent membrane release, was also resistant along with the mutant for Bro1p (Vps31p), which coordinates de-ubiquitination in the MVB pathway by recruiting Doa4p to endosomes [78]. Similar observations were made for Ni [32,79], while mutants lacking ESCRT proteins showed sensitivity to Cr, Cd and As. Interestingly, aside from ΔVps60, ESCRT mutants were sensitive to high Ca [32], which emphasizes the segregation of Ca and Gd by yeast, since ESCRT mutants were resistant to Gd and every other REEs (Figure 8).

Downstream the MVB, the retrograde transport is involved in the recycling of proteins and receptors of the plasma membrane or vacuolar membrane (through endosomes) to the trans-Golgi and/or back to the plasma membrane. Mutants for this process, together with other vesicular/endosomal transport, were also significantly enriched in our screen, with a Gd-sensitive phenotype. Firstly, the retrograde transport consists of one side of the GARP (Golgi-associated retrograde protein) complex, which is localized on the Golgi network. The GARP complex, which includes Vps51p, Vps52p and Vps54p [80], leads the retrograde transport of cytoplasmic vesicles from early endosomes to the late Golgi. Similarly to Ni, Cd, and chemical stressors [32], mutants for this function were sensitive to Gd. Secondly, this retrograde transport from the endosomal compartment to the late Golgi also involves the retromer complex on the endosome side. The latter complex consists of Vps5p, Vps17p, Vps29p, Vps35p, Mvp1p, Top1p and Pep8p. Mutants for components of the retromer complex were sensitive to Gd as they were for Cd [32].

It has previously been hypothesized that mutants for these different pathways (ESCRT and retrograde transport) could lead to protein missorting. Here, the disruption of the ESCRT complex, which occurs after membrane endocytosis and leads membrane degradation into the vacuole, increased yeast’s resistance to Gd. In contrast, a lack of the retrograde transport of endosomes to the Golgi and/or back to the plasma membrane leads to increased sensitivity. Therefore, a putative efflux system of Gd, localized in the plasma membrane, could remain at this location either by lack of endocytosis/degradation or by its recycling back to the plasma membrane. The accumulation of such transporter would contribute to Gd detoxification. Another consistent hypothesis could rely on endocytosis of Gd bound to the plasma membrane or engulfed into the lumen of endosomes. In both cases, intracellular Gd exposure and their subsequent toxic effects would be reduced. While these hypotheses seem valid, other indirect associated mechanisms cannot be ruled out and will require further attention.

### 3.10. Duality of the Sphingolipid Metabolic Pathway in Yeast Response to Gadolinium

Sphingolipids are a group of bioactive molecules that are involved in various cellular processes, including membrane fluidity and function, endocytosis, and signaling. We observed that sphingolipid biosynthetic and metabolic processes, as well as cellular sphingolipid homeostasis, were enriched in the mutant set analyzed (Figure 5, Supplementary Table S3). Interestingly, most of these mutants were resistant to Gd, while only a few showed sensitivity. Specifically, mutants that were disrupted for proteins involved in the early stages of sphingolipid synthesis, such as ΔOrm2, ΔElo2, ΔElo3 and ΔSur2, were resistant to Gd. These proteins are localized in the endoplasmic reticulum (ER) and catalyze the conversion of palmitoyl-CoA and serine into dihydrosphingosines (DHS) and ceramides. In contrast, mutants for enzymes implicated in the downstream steps of complex sphingolipid synthesis that occur in the Golgi, such as ΔSur1 and ΔCsg2, were sensitive to Gd. Our analysis of the sphingolipid synthesis pathway suggests the implication of complex sphingolipids and early simple sphingolipid forms (long-chain base, LCB) in the stress response to Gd and other lanthanides [49]. Previously, sphingolipid synthesis has been shown to mediate iron (Fe) toxicity in *S. cerevisiae*, with Orm2p playing a central role [81]. However, in our findings, the ∆Orm2 mutant was highly resistant to Gd, contrasting the sensitivity to high Fe concentrations. Therefore, Gd seems to modulate the sphingolipid-mediated response differently from Fe. Overall, our study provides insights into the role of sphingolipids in cellular responses to Gd stress, which may have implications in the development of strategies for controlling Gd exposure in various contexts.

## 4. Conclusions

While the number of reports mentioning Gd persistence in organisms after administration is increasing, the existing research on Gd cellular effects and homeostasis is limited, leaving open questions about how organisms can deal with this emerging contaminant. Our study used *S. cerevisiae* as a model organism to explore the molecular and cellular toxicity mechanisms together with the detoxification pathways involved in Gd stress response. We provide insights into the cell wall being the first interface against Gd, with endocytosis and plasma membrane transporters putatively regulating the uptake of Gd. Moreover, modifying the cell wall organization and the homeostasis of membrane sphingolipids appear to be key pathways in cellular response to Gd. Our study reveals possible general detoxification mechanisms for lighter lanthanides, such as vacuolar sequestration, that might not be conserved for heavier lanthanides. These findings provide a starting point for understanding Gd toxicity and can guide future investigations on more complex eukaryotes, including human cells. Several yeast genes playing pivotal role(s) in response to Gd stress are orthologs to human genes. Interestingly, these genes have been linked to renal dysfunction, bone density and ion homeostasis in humans, providing interesting leads to better understand the health risk and environmental impact associated with Gd. This study is therefore critical to improve risk assessment towards Gd and Gd-based MRI contrasting agents.

## Data Availability

The authors declare that all data related to the findings of this study are available within the article and supplementary information or are available from the corresponding author upon reasonable request.

## Supplementary Materials

The following supporting information can be downloaded at: https://www.mdpi.com/article/10.3390/microorganisms11082113/s1, Table S1: Detailed phenotypes and related information on the genes whose disruption affects gadolinium tolerance. Sensitivity and resistance levels were allocated to mutant strains as follows: low sensitivity (LS), medium sensitivity (MS) and high sensitivity (HS); and low resistance (LR), medium resistance (MR) and high resistance (HR). Table S2: Subcellular localization of the disrupted genes and proteins in mutant strains displaying a modified phenotype under gadolinium exposure. k is the number of mutants found in this study for each compartment, and f is the number of genes associated with a given compartment. NS = not significant. Table S3: GO-terms of biological pathways represented in sensitive and resistant mutants or sensitive and resistant mutants combined (All) with gadolinium. Table S4: GO-terms of molecular functions represented in sensitive and resistant mutants or sensitive and resistant mutants combined (All) with gadolinium.
